# Large Separable Kernel Attention–Driven Multidimensional Feature Cross-Level Fusion Classification Network of Knee Cartilage Injury: Algorithm Development and Validation

**DOI:** 10.2196/79748

**Published:** 2025-12-17

**Authors:** Lirong Zhang, Hang Yu, Yating Yang

**Affiliations:** 1 The School of Digital Art and Design, Dalian Neusoft University of Information, No. 8 Software Park Road, Ganjingzi District, Dalian, Liaoning, 116023, China, 86 13478427287

**Keywords:** knee cartilage injury, multilevel classification, large separable kernel attention, multidimensional feature, cross-level fusion

## Abstract

**Background:**

Knee cartilage injury (KCI) poses significant challenges in the early clinical diagnosis process, primarily due to its high incidence, the complexity of healing, and the limited sensitivity of initial imaging modalities.

**Objective:**

This study aims to employ magnetic resonance imaging and machine learning methods to enhance the classification accuracy of the classifier for KCI, improve the existing network structure, and demonstrate important clinical application value.

**Methods:**

The proposed methodology is a multidimensional feature cross-level fusion classification network driven by the large separable kernel attention, which enables high-precision hierarchical diagnosis of KCI through deep learning. The network first fuses shallow high-resolution features with deep semantic features via the cross-level fusion module. Then, the large separable kernel attention module is embedded in the YOLOv8 network. This network utilizes the combined optimization of depth-separable and point-by-point convolutions to enhance features at multiple scales, thereby dramatically improving the hierarchical characterization of cartilage damage. Finally, five classifications of knee cartilage injuries are performed by classifiers.

**Results:**

To overcome the limitations of network models trained with single-plane images, this study presents the first hospital-based multidimensional magnetic resonance imaging real dataset for KCI, on which the classification accuracy is 99.7%, the Kappa statistic is 99.6%, the *F*-measure is 99.7%, the sensitivity is 99.7%, and the specificity is 99.9%. The experimental results validate the feasibility of the proposed method.

**Conclusions:**

The experimental outcomes confirm that the proposed methodology not only achieves exceptional performance in classifying knee cartilage injuries but also offers substantial improvements over existing techniques. This underscores its potential for clinical deployment in enhancing diagnostic precision and efficiency.

## Introduction

**Figure 1.**
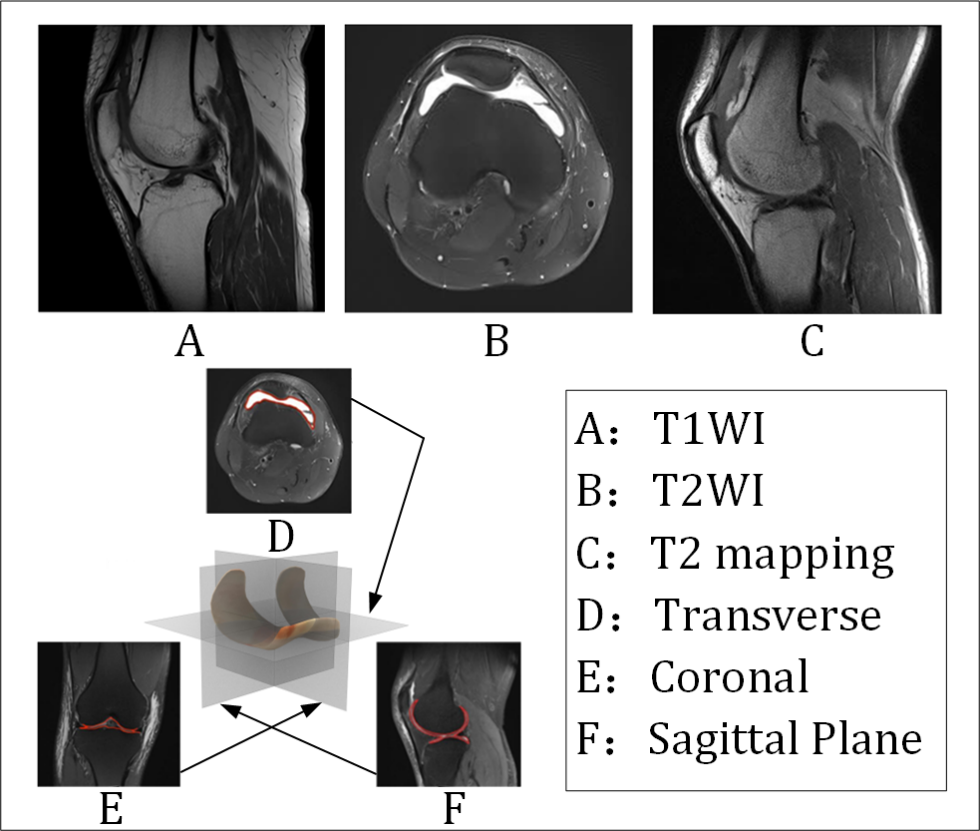
Multisequence and multiplane images of KCI in the retrospective study based on the clinical dataset provided by the Second Affiliated Hospital of Dalian Medical University, China. Multimodal KCI MRI images demonstrate T1WI, T2WI, and T2 mapping sequences across sagittal, coronal, and transverse planes. KCI: Knee cartilage injury; MRI: magnetic resonance imaging; T1WI: T1-weighted imaging; T2WI: T2-weighted imaging.

Knee cartilage injury (KCI) is recognized as a prevalent joint disease, with significant challenges posed to diagnosis and treatment by its high incidence and irreversibility [1]. The self-repair capacity of KCI is severely restricted due to the absence of vascularization and innervation. Consequently, the implementation of early and accurate diagnostic strategies has been established as critical for decelerating disease progression [2]. Currently, magnetic resonance imaging (MRI) is the preferred imaging tool for evaluating KCI due to its excellent soft tissue contrast. Clinically, MRI usually uses three orthogonal planes in the sagittal, coronal, and transverse planes for multiangle imaging. The sagittal plane displays the anatomical structures of the patellofemoral joint contact surface, the femoral trochanter groove, and the anterior and posterior corners of the meniscus. This is particularly beneficial for detecting early wear and tear and delamination injuries of the patellar and femoral trochanter cartilage [3]. The coronal plane facilitates observation of the integrity of the medial and lateral tibiofemoral joint surfaces and can visualize changes in cartilage thickness in weight-bearing areas of the tibial plateau and femoral condyles. It can also help identify the correlation between cartilage injury and adjacent bone marrow edema, while the transverse plane can accurately identify focal chondromalacia or fissures in the medial malleolar surface of the patella. Combined with multiplanar imaging, T1-weighted imaging (T1WI) [4], T2-weighted imaging (T2WI) [5], and T2 mapping [6] sequences can reflect KCI from multiple aspects, including structure, water content, and biochemical composition, as shown in Figure 1.

According to the International Cartilage Repair Society grading criteria [7], KCI is categorized into grades 0 to IV. Grade 0 indicates normal cartilage; Grade I shows surface softening and slight roughness of the cartilage; Grade II involves focal defects with a depth of less than 50% of the cartilage thickness; Grade III has a depth greater than 50% but not reaching the full thickness of the cartilage; and Grade IV represents the end-stage lesion with complete cartilage loss (Multimedia Appendix 1).

The clinical diagnosis of KCI has long depended on the subjective judgment of doctors on MRI images, which has problems such as low efficiency and lack of consistency [8]. With the development of computer vision technology, machine learning-based auxiliary diagnosis methods have gradually become a research hotspot [9].

However, the local receptive field of the fixed convolution kernel limits the capture of global context information. Lightweight models such as SqueezeNet [10] and MobileNet [11] reduce computational complexity through channel pruning and depth separable convolutions; however, they perform poorly when dealing with multisite and multistage cartilage damage. As the KCI area spreads or changes in thickness across multiple sites as the disease progresses, the aforementioned lightweight network models cannot provide optimal classification results. Current studies show that convolutional neural network (CNN) architectures still have insufficient feature representation ability for KCI classification tasks.

Recently, deep learning has made remarkable progress in the field of medical image analysis by virtue of its powerful feature learning ability. Traditional convolutional neural network architectures, such as AlexNet [12], ResNet [13], and GoogLeNet [14], enhance feature representation by increasing the network depth. However, the local receptive field of the fixed convolution kernel limits the capture of global context information. Lightweight models like SqueezeNet and MobileNet reduce computational complexity through channel pruning and depth separable convolutions, but they perform poorly when dealing with multisite and multistage cartilage damage. As the KCI area spreads or changes in thickness across multiple sites as the disease progresses, the aforementioned lightweight network models cannot provide optimal classification results. Current studies show that CNN architectures still have insufficient feature representation ability for KCI classification tasks.

To address the above limitations, researchers have proposed various improvement schemes. The multilayer convolutional sparse coding framework developed by Abdul et al [15] achieves hierarchical classification of ligament injuries through feature map connectivity modeling but still suffers from feature aliasing in complex lesion scenarios. The sample pyramid local binary pattern combined with K-nearest neighbor classifiers proposed by Demir’s [16] team optimizes the shallow texture feature representation, but it is difficult to overcome the interference of background redundant information in medical images. Double Attentive Graph Neural Network (DAGNet) adopts a directed acyclic graph architecture to achieve dynamic feature routing, but its higher computational complexity limits its application in small sample scenes [17]. Lei et al [18] employed convolutional neural network models such as DenseNet121, combined with data augmentation techniques, to achieve automatic classification of pressure ulcer stages. SgmaFuse [19] addresses interpretable breast cancer diagnosis in ultrasound imaging by integrating a spatially guided multilevel alignment mechanism and histological semantic activation vectors to bridge imaging features with pathological patterns. Dai et al [20] proposed a model enabling enhancement of breast cancer detection in mammographic images of dense tissue via a dynamic feature weighting Mamba module and edge-enhanced networks, reinforced by adversarial defense strategies for robustness. Rezk et al [21] developed a classification model that integrates image segmentation, skin tone clustering analysis, and style transfer, effectively enhancing the generalization capability of early skin cancer detection across all skin tones. Dual-stream parallel model based on local centroid optimization (LC-DSPNet) improves feature extraction capability through a localized center-of-mass optimization algorithm and a dual-stream parallel architecture, but it leads to partial loss of key lesion information, which affects the classification accuracy of complex knee cartilage injuries [22]. The improved AlexNet (R-AlexNet) combines the dragonfly optimization algorithm with the regional similarity transformation and achieves the current optimal performance in the task of KCI classification through multimodal feature fusion, but the limitation of its uniplanar dataset influences the model’s generalization ability [23]. The YOLO (You Only Look Once) series of algorithms is currently widely recognized in the field of computer vision for its efficient detection speed and excellent accuracy [24]. With the iterative optimization of the algorithms, the YOLO series has been extended from the target detection task to multiple vision task areas such as image classification and instance segmentation [25]. YOLOv8 significantly improves the model training efficiency and detection accuracy by introducing an adaptive learning rate adjustment mechanism and dynamic anchor frame generation technology [26]. Therefore, the YOLOv8 classification model is used as the baseline model in this paper to reduce the calculation complexity while maintaining the high precision classification ability and providing an effective means for the five-level classification of KCI.

Due to the low contrast and small target characteristics of MRI images of knee cartilage injuries, as well as background interference from bones and muscles, the traditional convolution module suffers from the defects of a large number of parameters and limited gradient flow, which make it difficult to effectively capture the fine lesion features, as well as the limitations of a single-plane dataset. For this reason, this paper proposes a multidimensional feature cross-level fusion (MDF-CLF) classification network driven by the large separable kernel attention (LSKA) for KCI, which reduces the computational complexity while maintaining the high-precision classification ability and provides a new solution for the five-level classification of KCI.

In summary, deep learning has shown significant potential in the field of medical image analysis [27], particularly in the automatic classification of knee cartilage, ligament, and meniscus injuries. However, current methods still face several key challenges: (1) single-plane MRI images struggle to fully capture the multidimensional features of KCI; (2) CNNs often lose the deep semantic information of small lesions during feature extraction; (3) the adaptability of multiscale feature fusion strategies is insufficient, limiting the accuracy of grading [28].

To address the above challenges, this paper proposes an MDF-CLF classification network driven by the LSKA. The purposes of this paper are as follows:

1. The first multidimensional KCI hospital real MRI data set is constructed: This study collaborated with the Second Affiliated Hospital of Dalian Medical University to collect a standard three-plane (sagittal, coronal, and transverse) knee MRI imaging. The T1WI, T2WI, and T2-mapping multiparameter sequences are integrated to create the first multidimensional imaging data set for KCI assessment, addressing the limitations of traditional single-plane medical imaging features.

2. An MDF-CLF network driven by LSKA has been constructed: The LSKA module is embedded into the YOLOv8 network, which integrates spatial feature extraction through deep separable convolution and channel information integration via point-bypoint convolution. This approach enhances multiscale features of KCI areas, effectively improving the feature representation capability for multilevel KCI.

The structure of this paper is organized as follows: Section 2 summarizes the related work; Section 3 introduces the network model proposed in this paper. Section 4 describes the image dataset, model training parameters, and experimental environment and analyzes the experimental results. Finally, Section 5 presents the conclusions and future directions of the algorithm.

## Methods

### Network Model Architecture

To address the limitations of traditional methods in knee cartilage damage classification, such as insufficient computational efficiency and limited ability to recognize subtle features, this paper proposes an MDF-CLF classification network algorithm driven by LSKA for KCI. The modules in the model proposed in this paper are described as follows.

**Figure 2.**
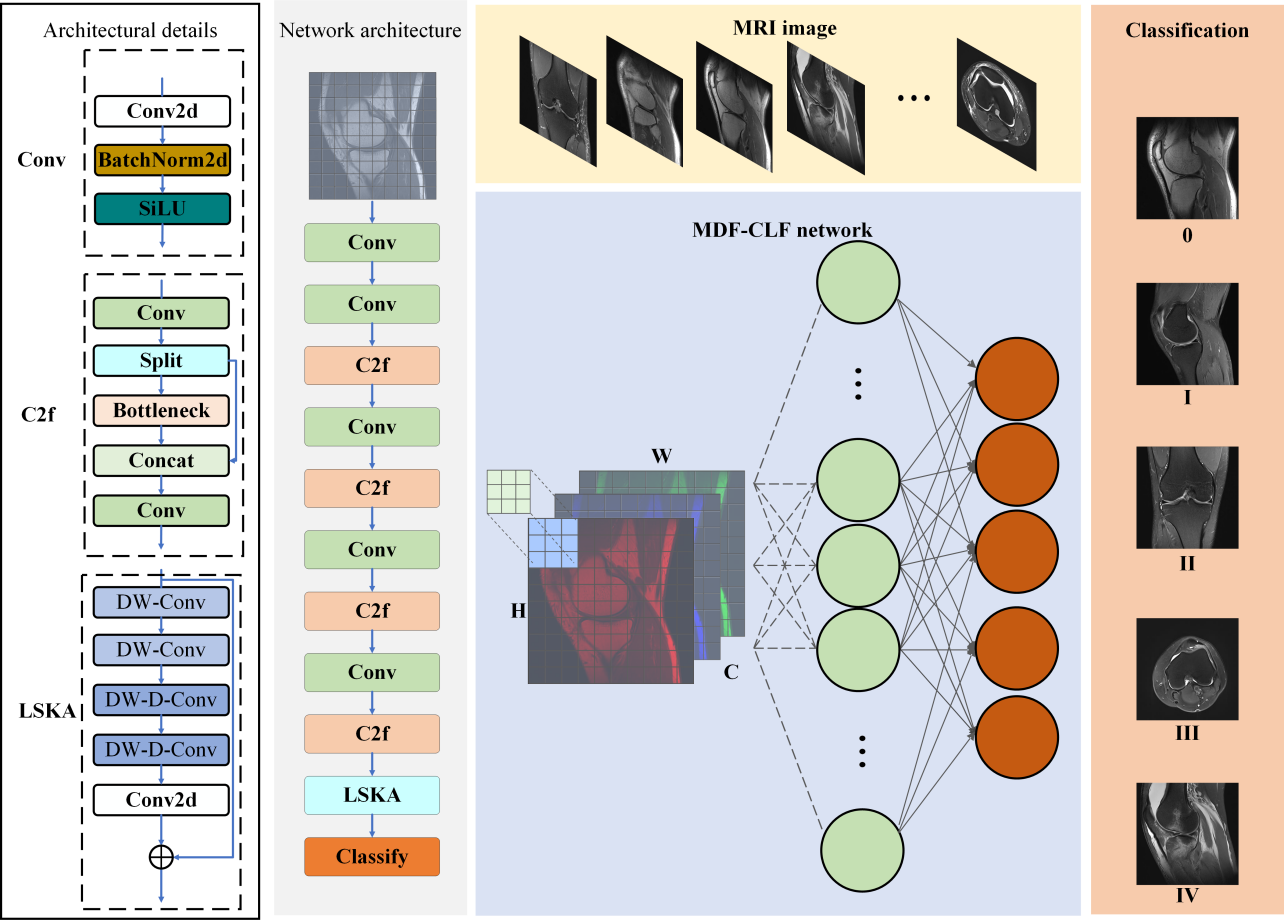
Flowchart of the proposed algorithm. Flowchart of the large separable kernel attention (LSKA)–driven multidimensional feature cross-level fusion (MDF-CLF) classification network, integrating LSKA, Cross-Stage Partial-Connection with 2 convolutions (C2f), and Conv modules within a YOLOv8-based framework, for 5-level knee cartilage injury classification using three-plane multimodal magnetic resonance imaging (MRI) images from a retrospective clinical cohort at the Second Affiliated Hospital of Dalian Medical University.

The algorithm introduces the LSKA module [29] into the backbone of the YOLOv8 framework. It constructs a feature extraction system composed of the Conv module [30], the C2f (Cross-Stage Partial-Connection with 2 convolutions) module [31], and the LSKA module and combines with the head network to realize attention focusing and multiscale feature fusion of the KCI region in MRI images. As shown in Figure 2, the network first inputs multidimensional MRI images of KCI into the feature extraction module. Here, the basic convolutional module uses the three-layer structure of the convolutional layer–batch normalization layer–SiLU (Sigmoid Weighted Linear Unit) activation function for primary feature extraction. It then realizes multilevel feature interaction and attention enhancement through the C2f module and the LSKA module. Finally, it completes the classification of the head network, which in turn effectively improves the accuracy and robustness of the classification of KCI.

### Conv Module

**Figure 3.**
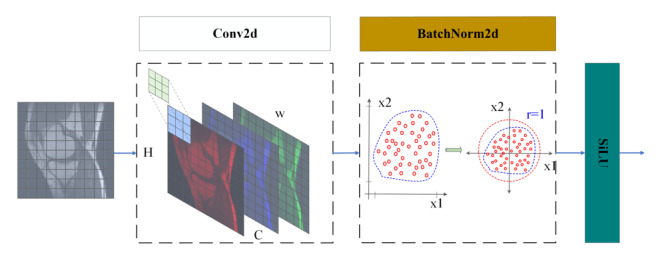
Conv module. Composition schematic of the Conv module integrating convolutional layer, batch normalization layer, and SiLU (Sigmoid Weighted Linear Unit) activation function in the LSKA-driven multidimensional feature cross-level fusion network for five-level classification of KCI based on real MRI data from the Second Affiliated Hospital of Dalian Medical University. KCI: knee cartilage injury; LSKA: large separable kernel attention; MRI: magnetic resonance imaging.

The Conv module is the basic and key component of this model, which consists of the convolution layer, batch normalization layer, and SiLU, as shown in Figure 3. The network model sets the input image as *I* ∈ *R^H×W×C^* , where *H* is the image height, *W* is the width, *C* is the number of channels, and the input image size is 640×640×3. The formula for convolution (Conv2d) can be expressed as:


(1) $$Y_{i,j}^{p}=\sum_{c=0}^{C-1}\sum_{m=0}^{N-1}\sum_{n=0}^{N-1}I_{i+m,\;j+n,\;c}\cdot K_{1}^{\;m,n,c,p}\;+\;b_{1}^{p}$$

where *i* and *j* are the positions of each pixel point in the feature map, *N* is the size of the convolution kernel, *m* and *n* are both indexes varying in the range of [0, *N−*1] used to traverse each value in the convolution kernel, and *p* is the index of the output channel (assuming that the number of convolution kernels is k, then 1≤*p*≤k). $Y_{i,j}^{p}$ represents the feature map output at channel *p*, position (*i*, *j*) after convolution in the first layer, and $I_{i+m,\;j+n,\;c}$ represents the input image *I* at channel *c*, the pixel value at position (*i+m*, *j+n*), $K_{1}^{\;m,n,c,p}$represents the convolution kernel of the first layer, and $b_{1}^{p}$ represents the bias term of channel *p* in the first layer.

To effectively suppress the internal covariate bias phenomenon during deep neural network training, the model introduces a batch normalization layer (BatchNorm2d). This module normalizes the feature maps output from the convolutional layer. It not only accelerates the convergence rate of the model but also enhances the stability of the training process by stabilizing the activation function input distribution.

Specifically, the normalization process of the BatchNorm2d layer takes the channel dimension as the unit of operation and assumes that the small batch sample capacity is *B*. For the *p*th channel, the normalization is performed for each element $Y_{ij}^{p}$ in the feature map $Y_{i,j}^{p}$:


(2) $${\hat{y}}_{ij}^{p}=\frac{y_{ij}^{p}-\mu^{p}}{\sqrt{\alpha_{p}^{2}+\varepsilon }}$$

where the mean $\mu^{p}=\frac{1}{B\cdot H_{\text{out}}\cdot W_{\text{out}}}\sum_{b=1}^{B}\sum_{i=1}^{H_{\text{out}}}\sum_{j=1}^{W_{\text{out}}}y_{ij}^{p}$, *∈* is a very small positive number, and variance $\alpha_{p}^{2}=\frac{1}{B\cdot H_{\text{out}}\cdot W_{\text{out}}}\sum_{b=1}^{B}\sum_{i=1}^{H_{\text{out}}}\sum_{j=1}^{W_{\text{out}}}y_{ij}^{l}(y_{ij}^{p}-\mu^{p}{)}^{2}$. *W*_*out*_ is the width of the output feature map and *H*_*out*_ is the height of the output feature map.

To recover the expressive ability lost during the normalization process and to make the model more flexible when learning data features, the module is optimized by introducing learnable scaling factors *γ*^*p*^ *∈ R* and level-shifting terms *β*^*p*^ *∈ R* into the backpropagation process. These terms are calculated as follows:


(3) $$y_{ij}^{p,BN}=\gamma^{p}{\hat{y}}_{ij}^{p}+\beta^{p}$$

To achieve a dynamic balance between model training stability and feature characterization capability, the module combines the probabilistic smoothing property of the sigmoid function with the conditional sparse activation advantage of the rectified linear unit function. This is calculated as follows:


(4) $$SiLU(x)=x\cdot \sigma (x)=\frac{x}{1+e^{-x}}$$

where *x* is the input feature and *σ*(*x*) is the sigmoid function.

The network architecture proposed in this paper realizes multiscale learning by adopting a hierarchical connection of convolutional modules. This enables the extraction of marginal features of the cartilage and texture features of the subchondral bone edema regions from the shallow network and the extraction of semantic features of KCI categories from the deep network. This allows for the accurate classification of KCI.

### C2f Module

**Figure 4.**
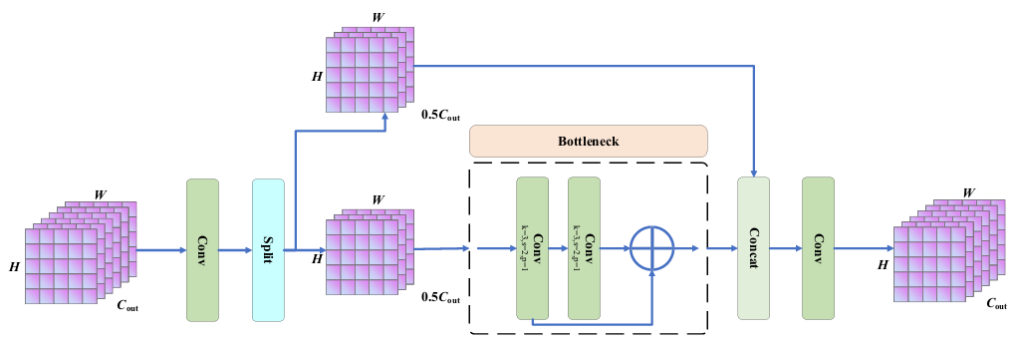
C2f module. Architecture of the C2f module implementing dual-path feature interaction with semantic enhancement path and detail preservation path for multiscale KCI classification in LSKA-driven multidimensional fusion network. C2f: Cross-Stage Partial-Connection with 2 convolutions; KCI: knee cartilage injury; LSKA: large separable kernel attention.

To address the contradiction between the attenuation of deep semantic feature information and the small-scale characteristics, this paper introduces a C2f module based on anatomical priors into the proposed network architecture. This module, through a dual-path feature interaction mechanism, provides robust support for the accurate identification of KCI, as shown in Figure 4. The dual-path design of the general-purpose C2f module is strategically adopted here because its inherent operational mechanism aligns effectively with the requirement to capture multiscale features of KCI, ranging from deep structural defects to subtle surface textural changes.

To effectively capture the multiscale damage feature information, it is divided into a semantic enhancement path *X*_1_ and a detail preservation path *X_2_* by cross-level fusion operation; first, the input feature map is divided into two parts according to the number of channels *C*. The expression of Split is as follows:


(5) $$\left(X_{1},X_{2})=Split(F,C_{1},C_{2}\right)$$

where *H×W×C_out_* is the dimension of the input feature map, *C*_1_ = *C*_2_ =0.5*C*_out_, and *C*_out_ is the number of channels in the output. The semantic enhancement *X*_1_ path processes features through Bottleneck units, which are dedicated to capturing the deep semantic information of knee joint deep structural injuries, including subchondral bone cystic lesions, full-thickness cartilage defects, and meniscus-cartilage junction injuries. It enhances the feature representation of such significant structural abnormalities through deep feature extraction, while mitigating gradient vanishing via residual design to improve the model’s ability to characterize moderate-to-severe KCI. *X*_1_ path uses the Bottleneck unit for deep extraction of features in the subchondral bone cystic lesion region and mitigates gradient vanishing by residual design to improve the sensitivity to the cartilage fissure. Concurrently, the detail preservation *X*_2_ path employs a skip connection to circumvent deep processing, thereby preserving high-resolution features essential for early injury identification. This pathway is specifically engineered to capture subtle alterations in the cartilage surface texture at the patellofemoral joint interface, minor abrasions in the meniscal anterior and posterior horns, and superficial cartilage defects within the tibial plateau weight-bearing regions. By mitigating the blurring effects induced by deep downsampling, this architectural choice furnishes critical feature support for precise early-stage KCI detection, enhancing the model’s responsiveness to nuanced pathological modifications in these anatomical locales. *X*_2_ path preserves the original feature details and mitigates the blurring of lesion edges caused by deep downsampling. Next, it is output through dual paths, and channel splicing is used to realize multiscale feature fusion.

At this time, the number of channels of the feature map becomes 0.5(*n*+2)*C*_out_ while the height and width remain as *H×W* , and *n*=1 is the number of Bottleneck cells. The expression of channel splicing is as follows:


(6) $$F_{Concat}=Concat(X_{1}^{out},X_{2})$$

where $X_{1}^{out}$ is the output of the *X*_1_ path, and *F*_*Concat*_ is the multiscale feature resulting from the splicing of the dual-path channels.

Finally, a 1×1 convolutional layer linearly transforms the number of channels to match the number initially convolved at input time, allowing the subsequent network structure to integrate seamlessly. In the C2f module, anatomical prior knowledge is integrated through a dual-path feature interaction mechanism, which orchestrates channel allocation and feature aggregation. The integration is achieved by leveraging the module’s inherent design, whereby the semantic enhancement path is utilized to capture features relevant to advanced, deep lesions, and the detail preservation path is harnessed to retain features essential for early, surface-level abnormalities. The semantic enhancement path extracts deep semantic features of knee cartilage injuries, while the detail preservation path maintains fine-grained features such as cartilage surface textures. Feature fusion is achieved via channel concatenation, enabling effective multiscale integration. Consequently, the C2f module incorporates knee joint anatomical priors through structured feature interaction and dynamic channel adjustment, effectively balancing semantic retention and detailed feature recognition to improve classification accuracy.

### LSKA Module

**Figure 5.**
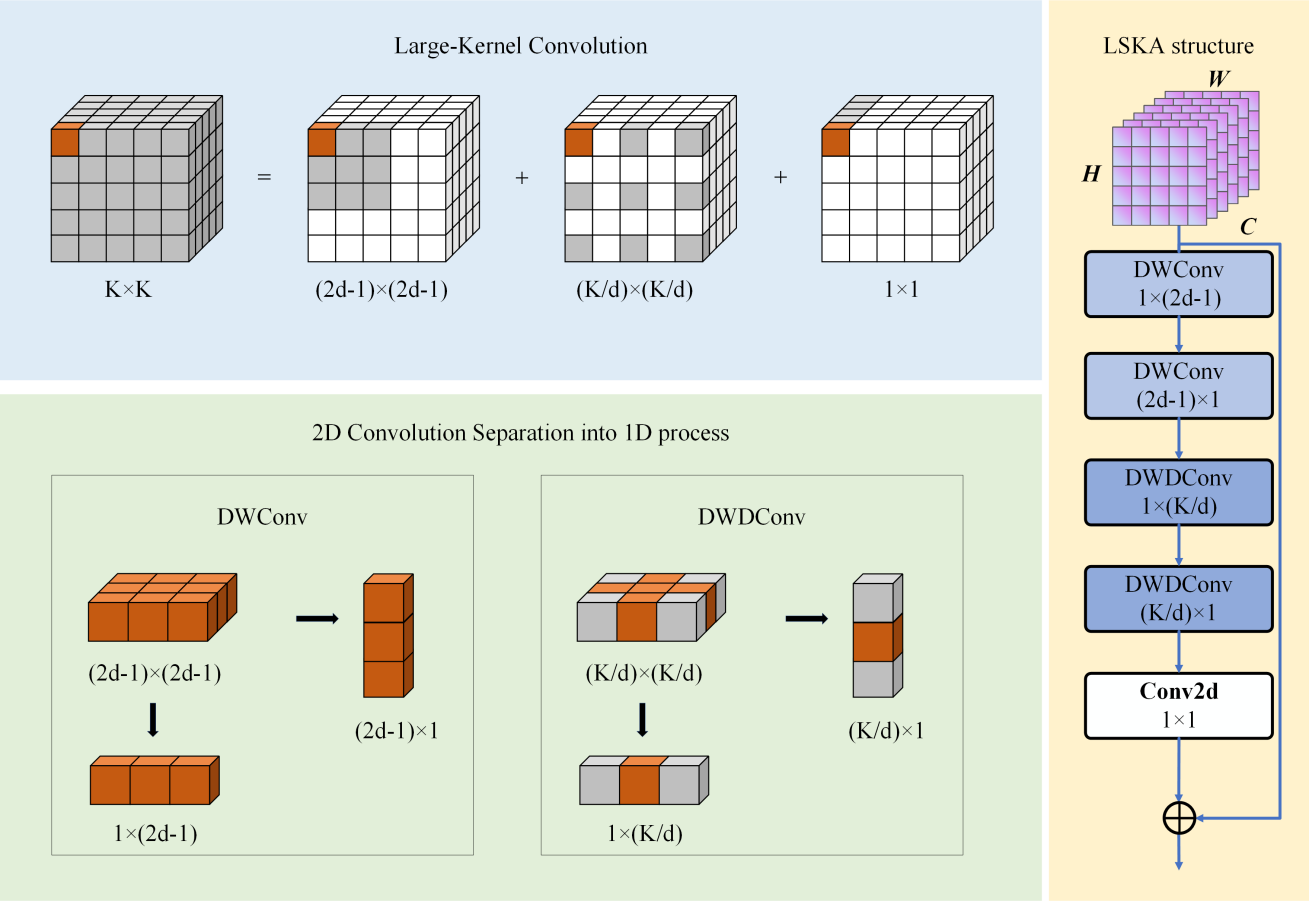
LSKA module structure diagram. Schematic of the LSKA module implementing Large-Kernel decomposition into depthwise convolution, dilated depthwise convolution, and point-by-point convolution for adaptive feature weighting in KCI classification network. KCI: knee cartilage injury; LSKA: large separable kernel attention.

To significantly enhance the cartilage fissure features and effectively improve classification accuracy, this paper introduces an LSKA module into the proposed network architecture. This module improves the sensitivity of long-distance cartilage, ligament, and tissue structures while maintaining computational efficiency through an efficient large kernel decomposition strategy with adaptive feature weighting. The network structure of the LSKA module is shown in Figure 5.

The LSKA decomposes the *k × k* convolution into depth convolution (DWConv), dilated depth convolution (DWDConv), and point-by-point convolution (PWConv).

To avoid the exacerbation of parameter redundancy due to the large size of the convolution kernel, DWConv extracts the spatial features independently for each channel and decomposes the traditional 2D depth convolution kernel *k × k* into a cascade of the horizontal convolution kernel (2*d* − 1) *×* 1 and the vertical convolution kernel 1 *× (2d − 1)* through the introduction of the dilatation rate *d*. The intermediate features afterward can be represented as:


(7) $${\bar{Z}}^{\;C}=\sum_{H,W}M_{1\times (2d-1)}^{C}{*}_{DW}\left(\sum_{H,W}M_{(2d-1)\times 1}^{C}{*}_{DW}F^{C}\right)$$

where *DW denotes the deep convolution operation, $M_{(2d-1)\times 1}^{C}$ and $M_{1\times (2d-1)}^{C}$ are 1D deep convolution kernels in the horizontal and vertical directions, respectively.

To maintain the sensitivity of the long-range structure of cartilage, ligaments, and other tissues, ${\bar{Z}}^{c}$ processes the intermediate features using dilation depth convolution. This decomposes the traditional 2D convolution kernel into horizontal and vertical 1D convolution kernels $1\times [\frac{k}{d}]$ and $[\frac{k}{d}]\times 1$, respectively, using the dilation rate *d*. The intermediate feature *Z*^*c*^ after DWConv can be expressed as follows:


(8) $$Z^{C}=\sum_{H,W}M_{1\times [\frac{k}{d}]}^{C}{*}_{\text{DW}}\left(\sum_{H,W}M_{[\frac{k}{d}]\times 1}^{C}{*}_{\text{DW}}{\bar{Z}}^{C}\right)$$

Besides, to achieve cross-channel information fusion, LSKA uses the 1 × 1 convolution kernel *M*_1 × 1_ to highlight the feature channels associated with knee cartilage damage. This kernel performs point-by-point convolution of the intermediate features *Z*^*c*^, which generates the attention map *A*^*c*^ expression:


(9) $$A^{C}=M_{1\times 1}{*}_{\text{PW}}Z^{C}$$

where *PW represents the pointwise convolution operation.

Finally, to enhance the features of the cartilage damage region of the KCI, the adaptive weighted feature map *F*^*c*^, which is generated by multiplying the attention map and the input feature map *F*^*c*^ element by element via the Hadamard product, can be represented as follows:


(10) $${\bar{F}}^{c}=A^{c}⊗F^{c}$$

Where ⊗ means element by element multiplication.

The LSKA module achieves adaptive feature weighting by identifying differences in the importance of cartilage injury-related features in knee joint MRI, dynamically allocating weights based on the contribution of features to injury identification, strengthening the representation of key injury features while suppressing background interference features. This mechanism exhibits significant advantages in distinguishing between mild surface softening and deep lesions; for the former, it can focus on enhancing the feature response of subtle texture changes on the cartilage surface to accurately capture early-stage injuries, while for the latter, it can prioritize strengthening the feature representation of deep cartilage structural abnormalities such as thickness defects and structural disruptions to avoid misjudgment of lesion depth effectively, achieving accurate differentiation between the two types of injuries.

### Classify Model

To map the features output by the backbone network to specific KCI categories, the head network module is used in this paper, whose structure consists of a fully connected layer and Softmax activation function.

After the backbone network embedded in the LSKA module processes the data, the feature vector *x_c_* is generated. This vector contains key information, such as soft tissue texture and semantics, from the knee MRI. To transform the high-dimensional features into injury categories, the head network undergoes linear transformation using the fully connected layer. This generates the response values of each injury category using the following mathematical expression:


(11) $$z=\eta x_{c}+b$$

where *η* is the weight matrix, *b* is the bias vector, *z* is the output vector of the fully connected layer, and the element *η*_*ij*_ in the weight matrix *η* denotes the extent to which the *j*th input feature contributes to the *i*th KCI output category.

Specifically, after being processed by the backbone network embedded with the LSKA module, a 1D feature vector *x_c_* is generated. This vector encapsulates key information from the knee MRI image, such as cartilage texture and semantic features. To map the high-dimensional features to specific damage categories, the head network utilizes a fully connected layer to perform a linear transformation, producing the response values for each damage category. Upon obtaining the output vector from the fully connected layer, the response value for the *o* knee cartilage damage category, based on the knee cartilage damage MRI image, can be acquired.

Based on the response value for the *o*th knee cartilage damage category from the knee cartilage damage MRI image, the confidence that the predicted knee cartilage damage MRI image belongs to the *o*th knee cartilage damage category is obtained. The formula used is as follows:


(12) $${\hat{y}}_{o}=\frac{e^{z_{o}}}{\sum_{o=1}^{5}e^{z_{o}}}$$

Where *ŷ*_*o*_ represents the confidence that the predicted knee cartilage damage MRI image belongs to the *o*th knee cartilage damage category; *z_o_* is the response value for the *o*th knee cartilage damage category based on the knee cartilage damage MRI image; *o* denotes the index of the knee cartilage damage category; and *e* is the base of the natural logarithm. The vector *z* is the output of the fully connected layer, containing the response values for the five knee cartilage damage categories, and *z_o_* is the response value corresponding to one specific category in *z*. The unnormalized class response values output by the fully connected layer cannot be directly used for classification decisions; they need to be transformed into a confidence distribution with probabilistic meaning. To achieve this, the Softmax activation function is applied for normalization.

In the five-class KCI classification task, for the *u*th sample (*u*=1, 2, ..., *U*) in the training set, where *U* is the total number of training samples, the output probability distribution from the LSKA-driven MDF-CLF classification network is denoted as ${\hat{y}}^{u}=({\hat{y}}_{1}^{u},{\hat{y}}_{2}^{u}\ldots ,{\hat{y}}_{5}^{u}{)}_{y}$. The formula for calculating the cross-entropy loss $L_{CE}^{u}$ of a single sample is as follows:


$$L_{CE}^{u}=-\sum_{o=1}^{O}y_{o}^{u}\log({\hat{y}}_{o}^{u})$$

(13) $$\sum_{o=1}^{O}{\hat{y}}_{o}^{u}=1$$

$L_{CE}^{u}$ represents the loss value of the *u*th KCI MRI sample; *O* denotes the index of the KCI category; *O* represents the total number of KCI categories. In this study, there are five categories of KCI. $y_{o}^{u}$ indicates the ground truth label of the *u*th KCI MRI sample, marking the true label value for the *o*th KCI category. ${\hat{y}}_{o}^{u}$ is the predicted probability that the *u*th KCI MRI sample belongs to the *o*th KCI category. In this study, the condition $\sum_{o=1}^{5}{\hat{y}}_{o}^{u}=1$must be satisfied to ensure the rationality and validity of the probability distribution. To prevent individual samples from interfering with the training, the mean loss of all samples in the training set is taken as the optimization objective. That is, the formula for the overall loss value *L* is:


(14) $$L=\frac{1}{U}\sum_{u=1}^{U}L_{CE}^{u}=\frac{1}{U}\sum_{u=1}^{U}\left(-\sum_{o=1}^{O}y_{o}^{u}\log({\hat{y}}_{o}^{u})\right)$$

where *L* represents the overall loss value of the cross-entropy loss function, *u* denotes the index of the KCI MRI sample, and *U* indicates the total number of KCI MRI samples. In this study, through backpropagation, parameters that increase the loss are adjusted along the negative direction of the gradient to mitigate the impact of misclassification, while parameters that decrease the loss are reinforced along the positive direction of the gradient. This process progressively enhances the model’s capability to identify low-contrast and small-scale lesion features, thereby achieving accurate classification of the five categories of injuries.

Finally, the parameters that increase the loss are adjusted in the opposite direction of the gradient to reduce the influence of misclassification through backpropagation. The parameters for reducing the loss are strengthened along the positive direction of the gradient, and the recognition ability of the model to low-contrast and small-scale features is gradually improved to realize the accurate classification of five types of damage.

### Experimental Environment and Datasets

All the experiments are implemented in PyTorch1.8, with Windows 10 system, Intel(R) Core(TM) with i7-9750H CPU and @ 2.60 GHz processor along with 16.0 GB of RAM, 64-bit Operating System, and NVIDIA GeForce RTX3090.

Besides, this study constructs a dataset based on real hospital data provided by the Second Affiliated Hospital of Dalian Medical University. This dataset comprises multisequence MRI images in three planes, which are sagittal plane, coronal plane, and transverse plane. Specifically, it includes 548 Grade 0 images, 674 Grade I images, 582 Grade II images, 539 Grade III images, and 579 Grade IV images. This dataset comprises 134 patients with KCI and 26 healthy individuals without knee pathology. Following patient-level data partitioning principles, all patients are randomly divided into two groups. Eighty percent of patients’ images are included in the training set, while the remaining 20% form a completely independent test set. Examples of images from the dataset are shown in Multimedia Appendix 1. During the acquisition of the multiparameter sequences involved in this study, which are T1WI, T2WI, and T2-mapping, strict adherence to clinical operating protocols ensures consistent spatiotemporal registration. All sequences are acquired on a 3.0T MRI scanner using identical field of view, matrix, slice thickness, and slice spacing parameters to guarantee spatial pixel correspondence across sequences. Following data acquisition, spatiotemporal registration is validated using anatomical landmarks of the knee joint, which include the anterior and posterior horns of the medial and lateral menisci and the femoral condyles. Additionally, image postprocessing software is employed to match and verify the coordinate points across all sequences, further ensuring consistency in spatiotemporal registration across the multiparameter sequences.

### Parameter Setting and Evaluation Index

To ensure the reasonableness of model training and testing, this paper divides the ratio of the dataset into training set and testing set according to 8:2, and the number of training rounds of the model is set to 350 rounds, with an initial learning rate of 0.01. The model with the highest accuracy on the test set is ultimately saved. During the training process, the stochastic gradient descent (SGD) optimizer dynamically adjusts to achieve efficient optimization.

To evaluate the performance of the proposed model, this paper employs several categorical evaluation metrics using accuracy (ACC), sensitivity (SEN), specificity (SPE), *F* measure (*F1*), and Kappa statistic (*K-S*) to assess the model’s performance, and these performance metrics are calculated as follows:


(15) $$ACC=\frac{TP+TN}{TP+TN+FP+FN}$$

(16) $$SEN=\frac{TP}{TP+FN}$$

(17) $$SPE=\frac{TP}{TP+FP}$$

(18) $$F1=\frac{2\times Sensitivity\times Specificity}{Sensitivity+Specificity}$$

(19) $$K\text{-}S=\frac{P_{0}-P_{e}}{1-P_{e}}$$

where $P_{o}=\frac{TP+TN}{TP+TN+FP+FN}$ , $P_{e}=\frac{\left(TP+FP)\times (TP+FN)+(FN+TN)\times (FP+TN\right)}{(TP+TN+FP+FN{)}^{2}}$. Besides, TP, TN, FP, and FN describe the number of true positives, true negatives, false positives, and false negatives. Here, the closer the values of ACC, SEN, SPE, *F1* , and *K-S* are to 1, the better the model performance. The *K*-*S* index is used to judge the difference between the degree of agreement of the classifier’s classification results and that of random classification.

Besides, this paper uses the confusion matrix to evaluate the performance of the classification model. The confusion matrix is a matrix that shows the relationship between the model predictions and the true labels. The rows of the matrix represent the true categories, the columns represent the predicted categories, and the values of the matrix elements indicate the number of samples for which a particular true category is predicted as each predicted category.

### Ethical Considerations

This study utilized retrospective and anonymized MRI data. In accordance with Article 32 of the Administrative Regulations on the Ethical Review of Biomedical Research Involving Humans (2023) [32] issued by the National Health Commission of China, ethical review approval is not mandatorily required for research utilizing exclusively deidentified archival data and records, provided that the use of such data does not pose any risk to the human subjects.

All MRI images used in this research are obtained from the Second Affiliated Hospital of Dalian Medical University. Prior to data collection and analysis, all personal identifiers are rigorously removed from the images and associated metadata to ensure complete patient anonymity, thereby mitigating any potential privacy risks. Furthermore, the hospital confirms that informed consent is obtained from all patients at the time of their initial clinical MRI scan. This consent process involved a comprehensive explanation of the nature of the imaging procedure and the potential future use of their deidentified data for research purposes, in line with the hospital’s standard operational protocols and in compliance with the Ethical Principles for Medical Research Involving Human Subjects outlined in the Declaration of Helsinki.

The authors hereby declare that the research is conducted in strict adherence to these principles and in compliance with all applicable local, national, and international guidelines and regulations concerning the protection of personal information and human rights, including China’s Personal Information Protection Law. The dataset construction and analysis procedures posed no additional physical or psychological risks to the patients involved.

## Results

### Results and Analysis

**Figure 6.**
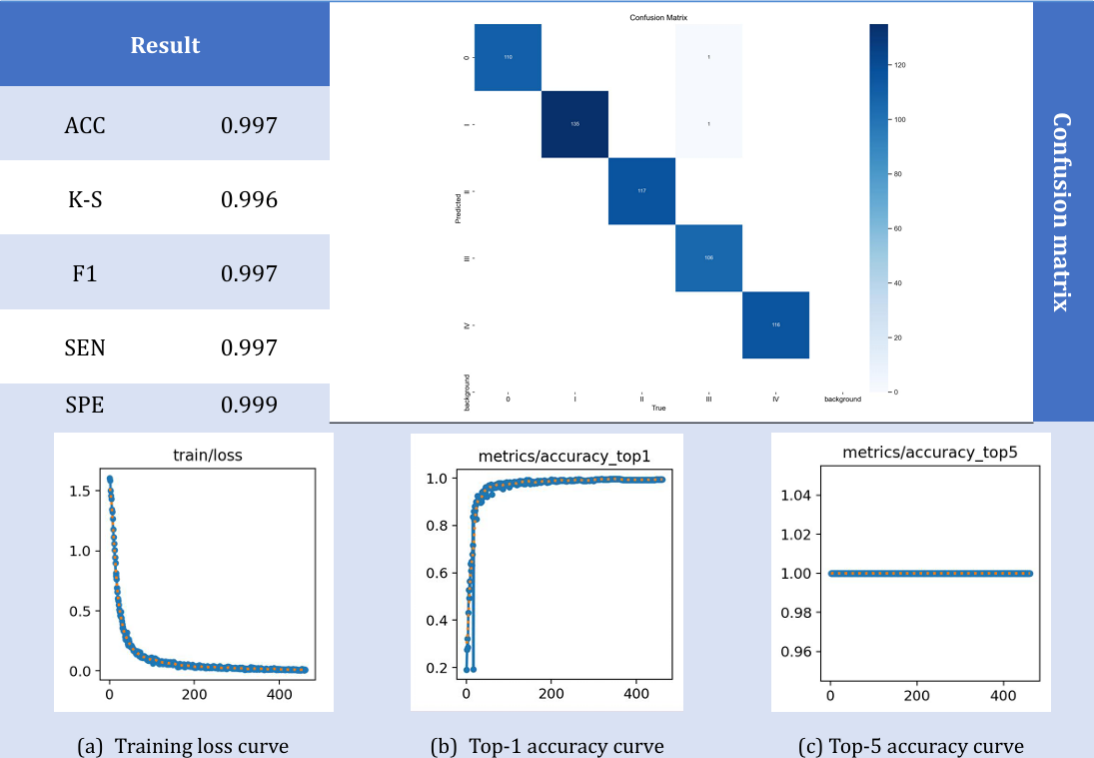
Experimental results of this algorithm. Performance evaluation of the LSKA-driven multidimensional feature cross-level fusion network for five-grade KCI classification using 3-plane multidimensional KCI MRI dataset from a retrospective clinical cohort at the Second Affiliated Hospital of Dalian Medical University, China: Achieving 99.7% accuracy with comprehensive metrics analysis. ACC: accuracy; F1: *F* measure; K-S: Kappa statistic; SEN: sensitivity; SPE: specificity.

The KCI classification algorithm proposed in this paper is tested on the constructed dataset to verify its performance. As shown in Figure 6, the algorithm achieved a classification accuracy of 99.7%, demonstrating its effectiveness. To verify the effectiveness of this algorithm, the following tests are carried out: different data set ratios, different training rounds, and different initial learning rates, as well as comparisons with other algorithms. The detailed data and analysis results are presented in the subsequent experiments.

### Experiment 1: Analysis of the Proportion Setting of the Dataset

**Figure 7.**
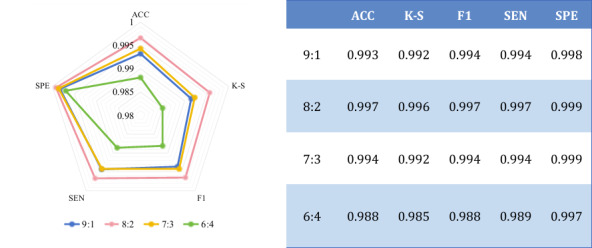
Experimental results of different proportion data sets. Ablation analysis of dataset partitioning ratios on the generalization performance of the LSKA-driven multidimensional feature cross-level fusion network for 5-grade KCI classification using 3-plane multidimensional KCI MRI dataset from a retrospective clinical cohort at the Second Affiliated Hospital of Dalian Medical University in China with optimal 82 split achieving 99.7% accuracy. ACC: accuracy; F1: *F* measure; K-S: Kappa statistic; SEN: sensitivity; SPE: specificity.

To assess the impact of data partitioning strategy on the model generalization ability, this paper designs multiple sets of ablation experiments in the constructed real hospital dataset and uses 6:4, 7:3, 8:2, and 9:1 partitioning ratios of the training and testing sets for comparative analysis, respectively. As shown in Figure 7, when 8:2 division is used, the model achieves the optimal balanced performance on the independent test set, with a classification accuracy of 99.7% and *F1* of 99.7%, which is significantly better than other ratios.

### Experiment 2: Analysis of the Epoch of Model Training

To observe the performance of the model proposed in this paper under different numbers of training rounds, this paper sets up multiple sets of controlled experiments, which are systematically tested by setting different numbers of training rounds. Under the condition that the ratio of the training set to the validation set is 8:2, the experimental results are shown in Multimedia Appendix 2. The data show that the early stopping mechanism is triggered when the model is trained for 461 rounds. It is worth noting that the accuracy of the network model peaks when the number of training rounds reaches 350. During the training cycle of 1 to 350 epochs, the model’s test set accuracy exhibited a steady upward trend, rising from 97.6% at 150 epochs to 99.7% at 350 epochs. When training proceeds to 400 epochs, the accuracy reaches 99.3%, and at 450 epochs, it is 99.5%, with a notable decline in test set accuracy observed. This phenomenon indicates that by 350 epochs, the model had fully learned the general features of the data and did not overfit to local noise in the training set due to overtraining. Meanwhile, the model design and training strategy established a multidimensional system to mitigate overfitting. At the data level, the multimodal T1WI, T2WI, T2 mapping, and multiplanar sagittal, coronal, transverse dataset characteristics effectively reduced the model’s dependence on single features, preventing the model from fitting only image noise under a single modality. At the optimizer level, the adopted stochastic gradient descent optimizer enabled dynamic learning rate adjustment, which automatically slowed the parameter update rate in the later training stage and effectively inhibited overfitting caused by aggressive parameter updates that fit local features of the training set. At the model structure level, the C2f module achieved dynamic balance in feature learning depth through dual-path feature interaction, namely the semantic enhancement path and the detail preservation path, further reducing the risk of overfitting.

### Experiment 3: Analysis of the Learning Rate for the Model Optimizer

**Figure 8.**
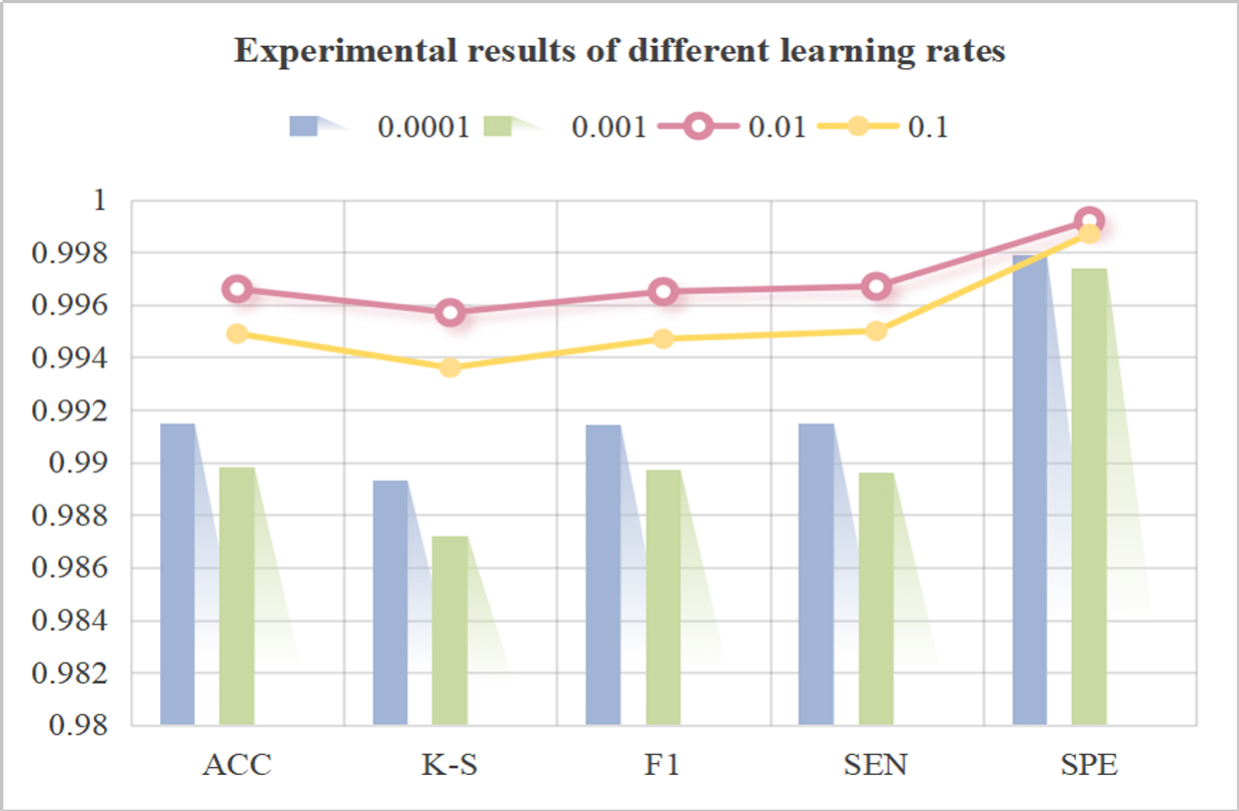
Experimental results of different learning rates. Analysis of optimizer learning rates in LSKA-driven multidimensional feature cross-level fusion classification network using 3-plane multidimensional KCI MRI dataset from the Second Affiliated Hospital of Dalian Medical University with optimal performance achieved by SGD at initial learning rate 0.01. ACC: accuracy; F1: *F* measure; K-S: Kappa statistic; SEN: sensitivity; SPE: specificity.

To explore the optimal parameter configuration of the improved algorithm proposed in this paper to achieve the best training effect, this paper adopts the SGD optimizer and systematically carries out comparative experiments under the conditions of different initial learning rates. As shown in Figure 8, the experimental results show that under the premise of using the SGD optimizer, the initial learning rate has a large impact on the model training effect. When the initial learning rate is set to 0.01, the network model shows the optimal performance index in the validation set, which realizes the best training effect under the current experimental conditions and provides a scientific and reasonable basis for setting parameters for subsequent model training.

### Experiment 4: Comparison With the State-of-the-Art Algorithms

To objectively and comprehensively evaluate the performance of the algorithm proposed in this paper, it is compared and experimented with a number of representative classical and representative models in the current field, and all the models are trained based on the same dataset to ensure reliable results. Table 1 shows the comparison data of various indexes.

**Table 1.** Comparison results with the state-of-the-art algorithms^a^.

Method	ACC	K-S	F1	SEN	SPE	Test times
AlexNet	0.915	0.893	0.914	0.918	0.979	9.0067
ResNet	0.889	0.862	0.890	0.894	0.973	20.2901
GoogLeNet	0.924	0.904	0.924	0.924	0.981	8.4832
SqueezeNet	0.895	0.869	0.896	0.898	0.974	10.9536
MobileNet	0.859	0.823	0.859	0.864	0.965	15.9256
DAGNet^b^	0.986	0.983	0.986	0.986	0.997	20.4942
R-AlexNet	0.941	0.926	0.940	0.940	0.985	9.0759
LC-DSPNet^c^	0.874	0.843	0.870	0.871	0.969	7.0432
YOLOv8	0.988	0.985	0.988	0.988	0.997	0.0024
MDF-CLF	0.997	0.996	0.997	0.997	0.999	0.0025

a Comparison of the LSKA-driven multidimensional feature cross-level fusion (MDF-CLF) network with state-of-the-art algorithms on the 3-plane multidimensional KCI MRI dataset from the Second Affiliated Hospital of Dalian Medical University, demonstrating MDF-CLF achieves optimal performance across all evaluation metrics.

b DAGNet: Double Attentive Graph Neural Network.

c LC-DSPNet: Dual-stream parallel model based on local centroid optimization.

The results show that the algorithm proposed in this paper performs outstandingly in the task of classifying five levels of KCI and outperforms the comparison models in all indicators. Its accuracy rate reaches 0.997, which can correctly determine the injury category, and it is reliable in clinical application and can provide a strong reference for diagnosis. The model features a compact architecture with 1,515,605 parameters. Training efficiency is validated on a single NVIDIA RTX 3090 GPU, where completing 350 epochs takes 5.99 hours, averaging approximately 62 seconds per epoch, demonstrating efficient training capabilities. More critically, the inference speed measured on the same hardware during validation reached 2.5 ms per image, equivalent to a throughput of 400 images per second. These empirical results demonstrate that the model achieves state-of-the-art classification accuracy while maintaining a low computational burden and high inference efficiency, supporting its potential for clinical applications.

### Experiment 5: Ablation Analysis of Individual and Combined Imaging Plane Contributions

A comprehensive ablation study is conducted to evaluate the diagnostic value of individual anatomical imaging planes and their combined performance in classifying knee cartilage injuries. The experimental methodology involved a comparative analysis of model performance when trained exclusively on single-plane data in sagittal, coronal, and transverse orientations versus the complete triple-plane combination. The results of our experiments are illustrated in Multimedia Appendix 3. The results demonstrate that the sagittal plane achieved the best single-plane performance with 99.6% accuracy, attributable to its exceptional capability in visualizing critical anatomical structures, such as the patellofemoral joint contact surface and anterior/posterior meniscal horns, which are essential for detecting early-stage cartilage wear and delamination injuries. The coronal plane yielded 99.3% accuracy, effectively capturing tibiofemoral joint integrity and cartilage thickness variations in weight-bearing regions, while the transverse plane achieved 99.1% accuracy, confirming its value in identifying focal chondromalacia and fissures on the medial malleolar surface of the patella. Integration of all three anatomical planes achieved an optimal accuracy of 99.7%, representing a 0.1% improvement over the sagittal-plane-only approach; the multiplane fusion strategy inherently enhances robustness through redundancy. In clinical practice, individual MRI planes may be compromised by artifacts, noise, or acquisition inconsistencies; the fusion strategy mitigates such risks by leveraging complementary information across planes. Additionally, features from the sagittal plane enhance contextual understanding of coronal and transverse data, promoting a more comprehensive feature representation and highlighting the synergistic potential of triple-plane integration.

## Discussion

### Principal Findings

In response to the growing demand for accurate medical imaging diagnostics, this study focuses on KCI classification by proposing an MDF-CLF network guided by the LSKA mechanism. The network integrates sagittal, coronal, and axial MRI images to construct a 5-class KCI dataset, achieving a final accuracy of 99.7%. The innovative optimization of the YOLOv8 backbone architecture enhances feature extraction capabilities, enabling precise localization of injury regions and identification of subtle lesion characteristics. The model’s performance has been rigorously validated on real-world hospital datasets, demonstrating strong potential for supporting clinical decision-making.

### Comparison to Prior Work

Unlike traditional approaches that often rely on single-plane imaging or limited feature integration, our method leverages multimodal MRI data and incorporates an LSKA-based attention mechanism to improve contextual feature capture. While previous studies have utilized deep learning for KCI classification, few have achieved comparable accuracy through multiplane fusion and attention-guided localization. Our model outperforms existing methods in both precision and generalizability, addressing a critical gap in prior work, which often overlooked the integration of cross-plane features and scalable attention mechanisms.

### Limitations

This study aims to enhance the classification accuracy of KCI diagnosis by proposing a MDF-CLF classification network driven by LSKA. The method integrates the LSKA module into the YOLOv8 framework, combined with the C2f module and convolutional base modules, achieving multiscale feature enhancement and semantic information extraction from multimodal MRI images. However, although the dataset is derived from real clinical MRI data, all data are acquired from a single medical center using uniform 3.0T MRI scanning equipment and consistent imaging parameters. While this homogeneity ensures internal data consistency, it may constrain the model’s generalization capability, potentially leading to performance degradation when applied to heterogeneous data from different centers or equipment. To address this limitation and enhance the model’s robustness, future work will employ federated learning techniques to integrate multicenter data. This strategy is expected to significantly improve the model’s generalization performance, laying a solid foundation for the broad clinical application of KCI diagnostic models.

### Future Directions

Future research will focus on enhancing the algorithm’s accuracy and efficiency through lightweight architectural design and cross-domain adaptation, expanding its application to other medical imaging domains such as ocular, pulmonary, and renal diseases, which share analogous challenges in fine-grained classification, and exploring integrated multidisease diagnostic frameworks to broaden the clinical impact and generalizability of the proposed methodology.

### Conclusions

This study achieves its initial goal of developing a high-accuracy deep learning model for KCI classification using multimodal MRI fusion and attention mechanisms. The proposed MDF-CLF network not only facilitates precise knee cartilage diagnosis but also provides a transferable framework for other medical imaging classification tasks. By addressing key limitations and outlining clear future directions, this work contributes to advancing automated diagnostic technologies and supports their integration into clinical practice.

## Supplementary material

10.2196/79748 Multimedia Appendix 1 Example image of the dataset.

10.2196/79748 Multimedia Appendix 2 Experimental results of different training epochs. Experimental analysis of training epochs on the performance of the large separable kernel attention–driven multidimensional feature cross-level fusion network for 5-Grade knee cartilage injury (KCI) classification using 3-plane multidimensional KCI magnetic resonance imaging dataset from a retrospective clinical cohort at the Second Affiliated Hospital of Dalian Medical University in China with optimal accuracy of 99.7% at 350 epochs.

10.2196/79748 Multimedia Appendix 3 Comparison results with the plane ablation experiment. Plane ablation experiment comparison in large separable kernel attention–driven multidimensional feature cross-level fusion classification network using 3-plane multidimensional knee cartilage injury magnetic resonance imaging dataset from the Second Affiliated Hospital of Dalian Medical University with multiplane fusion achieving optimal classification accuracy.

## Abbreviations

C2f: Cross-Stage Partial-Connection with 2 convolutions
CNNs: convolutional neural networks
DAGNet: Double Attentive Graph Neural Network
KCI: knee cartilage injury
LC-DSPNet: Dual-stream parallel model based on local centroid optimization
LSKA: large separable kernel attention
MDF-CLF: multidimensional feature cross-level fusion
MRI: magnetic resonance imaging
SiLU: Sigmoid Weighted Linear Unit
T1WI: T1-weighted imaging
T2WI: T2-weighted imaging
YOLO: You Only Look Once
