# Supplementary figures and images for: Large Separable Kernel Attention–Driven Multidimensional Feature Cross-Level Fusion Classification Network of Knee Cartilage Injury: Algorithm Development and Validation
Source: JMIR Med Inform. 2025 Dec 17;13:e79748. doi: 10.2196/79748 (PMC12711134; doi:10.2196/79748)

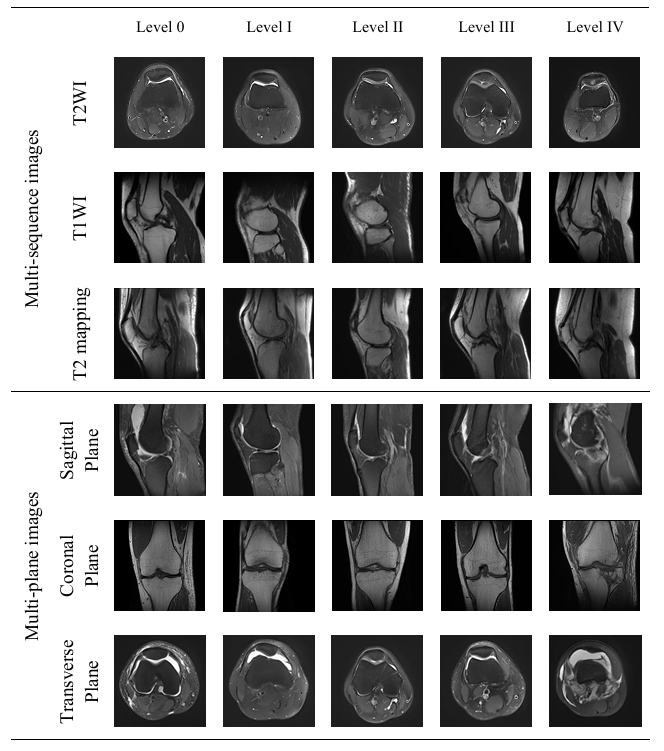

Supplement: Multimedia Appendix 1 [file medinform-v13-e79748-s001.png]

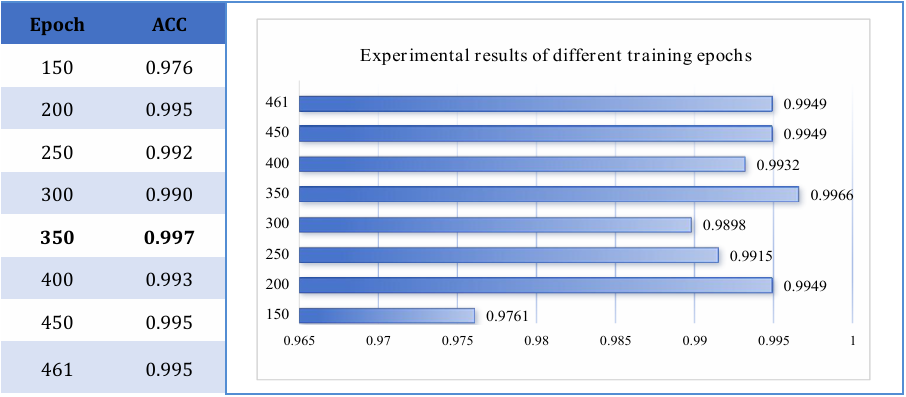

Supplement: Multimedia Appendix 2 [file medinform-v13-e79748-s002.png]

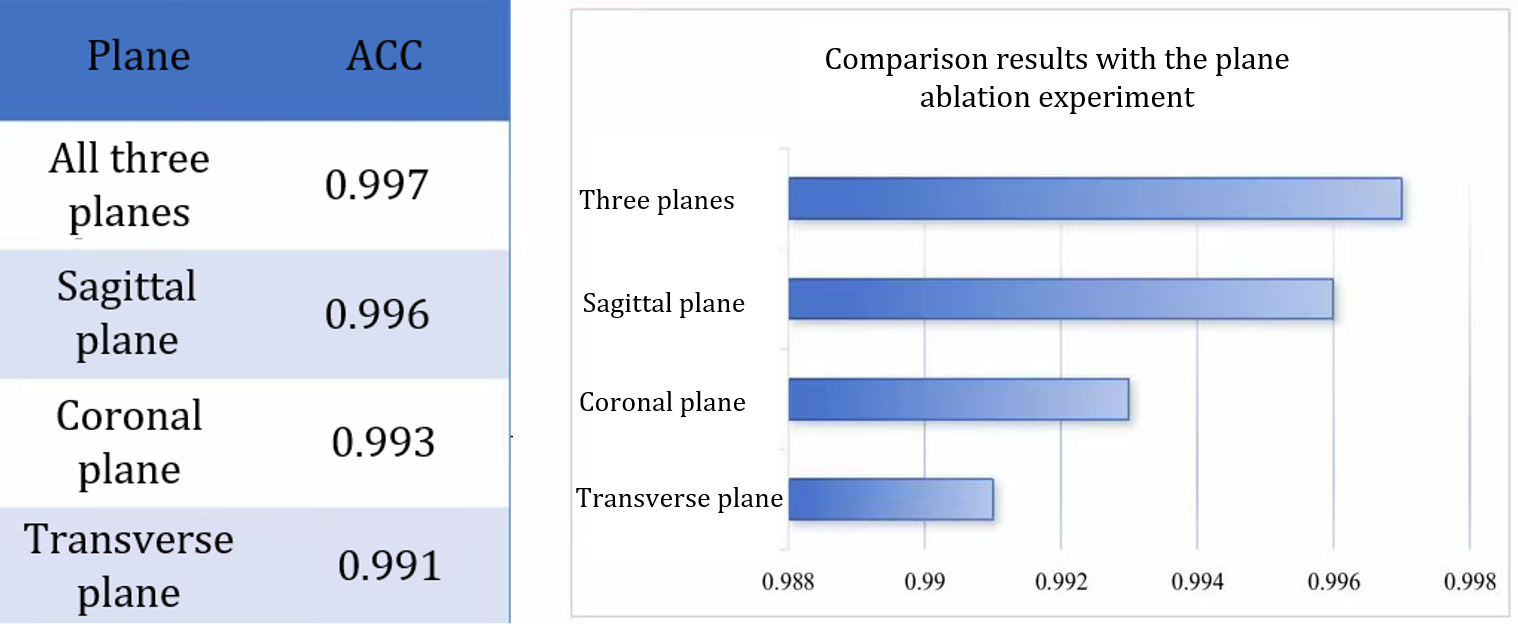

Supplement: Multimedia Appendix 3 [file medinform-v13-e79748-s003.png]
